# Channel Modeling for Multi-Receiver Molecular Communication System by Impulsive Force in Internet of Nano Things

**DOI:** 10.3390/s25113472

**Published:** 2025-05-30

**Authors:** Pengfei Zhang, Pengfei Lu, Xuening Liao, Xiaofang Wang, Ping Zhou

**Affiliations:** 1 College of Information Science and Technology, Shihezi University, Shihezi 832003, China; 20232108002@stu.shzu.edu.cn (P.Z.); xfwang@shzu.edu.cn (X.W.); ping_0626@hotmail.com (P.Z.); 2 School of Computer Science, Shaanxi Normal University, Xi’an 710062, China; liaoxuening@snnu.edu.cn; 3 Shaanxi Key Laboratory for Network Computing and Security Technology, Xi’an 710048, China

**Keywords:** molecular communication, internet of nano things, channel modeling, initial pulse, single-input multiple-output, multiple receivers

## Abstract

When studying molecular communication (MC) systems within fluid environments of the Internet of Nano Things (IoNT), fluid resistance has a significant impact on molecular transmission characteristics. In single-input multiple-output (SIMO) scenarios with multiple receivers, the interaction between fluid effects and inter-receiver interference complicates the modeling process. To address these challenges, this paper incorporates fluid resistance into a three-dimensional SIMO model and investigates the impact of the angle between receivers and the direction of the molecular pulse—considering both azimuth and polar angles—on the number of molecules received. Additionally, the interference from other receivers on the primary receiver is analyzed, and a mathematical expression for the number of received molecules is derived. Simulation results validate the model’s accuracy. The experiments show that as the distance between the interfering receiver and the transmitter increases from 0.10 m to 0.95 m, the number of molecules received by the primary receiver first rises and then falls, exhibiting a nonlinear interference pattern. Moreover, reception efficiency peaks when the receiver is positioned at a polar angle of 90° and an azimuth of 0°, with deviations from these angles leading to performance degradation. The spatial arrangement of receivers and transmitters, the number of receivers, and the initial velocity of molecules all significantly influence reception performance.

## 1. Introduction

**Figure 1:**
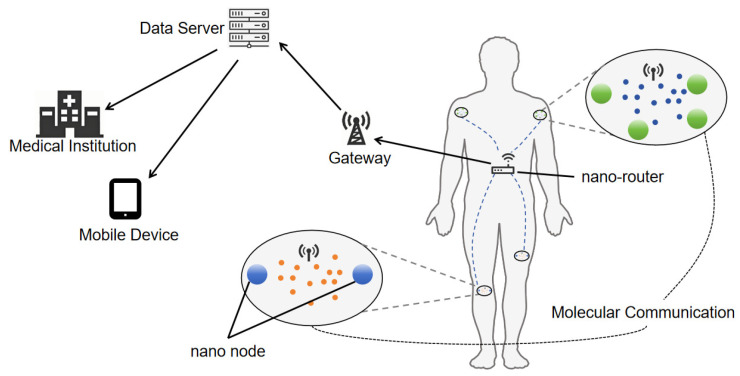
IoNT network architecture.

With the rapid development of nanotechnology, nano nodes have attracted widespread attention as emerging information units. Nano nodes can be either nanomachines or biological nanosensors. Since individual nano nodes are only capable of performing basic sensing and computational tasks, they need to communicate and cooperate with each other to address more complex requirements. This mode of collaboration has led to the formation of the Internet of Nano Things (IoNT), as illustrated in Figure 1 [1,2,3].

**Figure 2:**
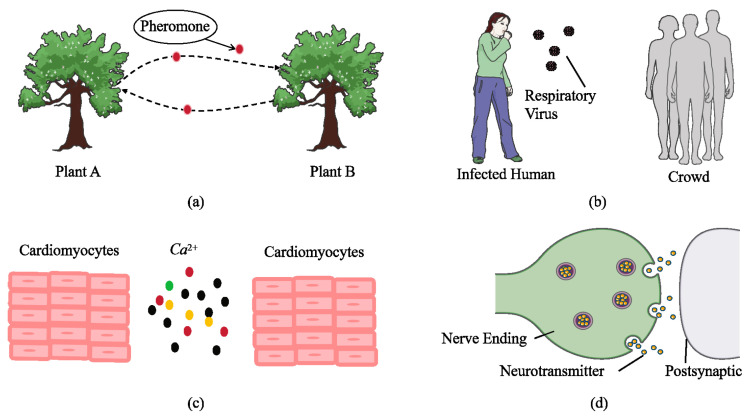
Various scenarios of molecular communication. (**a**) Pheromone transmission between plants. (**b**) Infected individuals spreading respiratory viruses to the crowd through coughing. (**c**) Cardiomyocyte communication. (**d**) Nerve endings releasing neurotransmitters. The figure is adapted from an existing image provided by Servier Medical Art by Servier, licensed under a Creative Commons Attribution 3.0 Unported License.

Molecular communication (MC) stands out as a highly promising communication method for the IoNT due to its efficiency at the nanoscale and its high compatibility with biological systems [4,5]. Unlike traditional electromagnetic communication, MC employs molecules carrying encoded information that diffuse through a medium, undergo chemical reactions, and utilize other biological mechanisms to transmit signals [6,7,8]. This facilitates biological compatibility and seamless interaction with naturally occurring biological signals [9,10,11]. MC has a wide array of applications, having demonstrated significant potential in scenarios such as cellular signal analysis, real-time ecological monitoring, and public health (e.g., investigating virus transmission mechanisms), as shown in Figure 2 [12,13].

The majority of existing MC research is based on the single-input single-output (SISO) model, mainly focusing on fundamental propagation characteristics between a single transmitter and a single receiver [14,15]. However, the SISO architecture has significant limitations in capturing spatial heterogeneity and interference in multi-receiver scenarios, making it difficult to adequately represent complex real-world communication environments [16]. For example, in cancer therapy, nanorobots acting as drug carriers may simultaneously release anticancer drugs to multiple tumor cells, thereby enhancing drug targeting and therapeutic efficacy [17]. Meanwhile, immune cells communicate by secreting cytokines; a signal molecule released from one immune cell can be detected by multiple others, regulating immune responses [18]. In such scenarios, the influence of the surrounding fluid medium on molecular transport, the relationship among multiple receivers, and higher-dimensional spatial factors make SISO models insufficient for multi-receiver communication networks.

To address these challenges, researchers have begun exploring single-input multiple-output (SIMO) MC systems, which more accurately characterize signal propagation dynamics in multi-receiver environments. Particularly, pulse-assisted techniques have enhanced the capability of multiple receivers to simultaneously capture signals, significantly improving information acquisition efficiency and interference robustness [9,19,20,21]. SIMO systems have demonstrated strong applicability in modeling viral transmission, ecological monitoring, and precise drug delivery, positioning them as a key direction for future MC research and applications. Khalid et al. investigated fluid-assisted SISO MC models, studying aerosol transmission in respiratory virus spread [13,22]. Huang et al. divided molecular transport into three stages, analyzing fluid-assisted transport and free-diffusion separately [23].

Nevertheless, current studies face two major constraints. First, the majority of existing work focuses primarily on SISO scenarios and lacks a systematic analysis of molecular propagation in multi-receiver environments. Second, most SIMO-related studies concentrate on two-dimensional settings; although some address three-dimensional scenarios, they often reduce dimensionality back to two dimensions [24]. Moreover, research on SIMO models has not yet extended to environments with fluid velocity. To overcome these issues, this paper models and analyzes a three-dimensional SIMO MC system under pulse-assisted conditions. Specifically, we account for various factors affecting molecular propagation in a multi-receiver environment, including the distance between transmitter and receivers, spatial angles (azimuth and polar angles), and the impact of the initial pulse. Table 1 highlights the main distinctions between this study and previous models. Our work addresses the following shortcomings in existing models: (1) It moves beyond the focus on predominantly two-dimensional spaces in SIMO studies, extending to a three-dimensional setting that is more reflective of real-world scenarios. (2) It extends the consideration of fluid-assisted factors from SISO systems to SIMO environments, particularly by introducing pulsed airflow in a fixed direction. (3) Under conditions involving fluid velocity, the model accounts for mutual interference among multiple receivers.

The main contributions of this article are as follows:

We analyzed the channel response of the SIMO MC model in three-dimensional scenarios, taking into account factors such as convection velocity, resistance, receiver positioning, and interference effects between receivers. We validated the model’s effectiveness through convection–diffusion equation modeling and numerical simulations.

1. In the SISO model, we considered convection velocity and resistance factors, modeling and analyzing them using theoretical approaches such as the convection–diffusion equation and differential equations. This process enhanced the model, enabling it to more accurately describe the behavior of molecular propagation in the channel, particularly the diffusion characteristics of molecules under the influence of a fluid medium.

2. We extended the SISO model to a SIMO model and conducted an in-depth study of the SIMO MC model’s channel response in three-dimensional scenarios. We focused on analyzing the impact of the receiver’s positional factors on the channel response, including the distance and angle between the transmitter and receiver. The primary analysis explored the relationship between these factors and the system response, providing insights for optimizing receiver layouts in practical applications.

3. We quantified the interference effects between receivers in the SIMO model, further enhancing the accuracy of channel response in such models. By modeling the interference process of interfering receivers on the primary receiver and proposing the corresponding system response expression, the model is able to more comprehensively reflect the molecular propagation characteristics in multi-receiver scenarios.

4. The system performance was studied through numerical simulations, and the proposed SIMO MC model in three-dimensional space was evaluated. A series of simulation experiments demonstrated the impact of factors of interest (such as distance, elevation angle, and azimuth angle) as well as interference between receivers on the number of molecules received by the receivers in the SIMO system, thereby establishing the model’s validity and accuracy in this scenario.

The structure of this paper is arranged as follows. Section 1 introduces the background and research motivation of MC. Section 2 presents the pulse-assisted SIMO MC system model. Section 3 details the channel modeling methods and mathematical derivations. Section 4 provides numerical results and analyzes the impact of key parameters on system performance. Section 5 discusses the research. Section 6 offers conclusions of the study.

## 2. System Model

**Figure 3:**
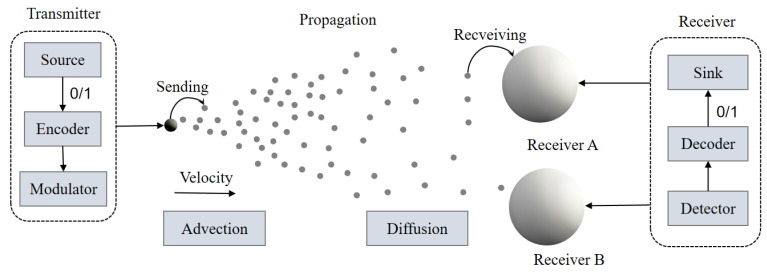
Framework diagram of a SIMO MC system. The system consists of a transmitter, a propagation medium, and a receiver. The transmitter converts binary signals (0/1) into molecular signals through an encoder and a modulator, and transmits the molecules in the propagation medium via advection and diffusion mechanisms. The receiver includes multiple receptors (e.g., Receiver A and Receiver B), which detect and decode the signals through detectors and decoders, ultimately outputting binary data.

This study proposes a three-dimensional SIMO MC system model, consisting of a transmitter, a channel, and multiple receivers (as illustrated in Figure 3). The transmitter acts as the signal source, instantaneously releasing information-carrying molecules that propagate through the air channel. The trajectories and velocities of these molecules are influenced by fluid dynamics and are ultimately captured by the receivers. The spatial arrangement of receivers significantly affects their performance; this paper further investigates how receiver deployment parameters influence the number of molecules received and the overall system performance.

The channel serves as the medium for molecular propagation. In biological systems, molecular signals such as hormones and neurotransmitters are transmitted across cell membranes or intercellular gaps [25,26]. In environmental monitoring, sensors detect changes in airborne molecular concentrations to track pollutants. In drug delivery systems, drug molecules diffuse from biomaterials into bodily fluids, with the diffusion pathway constituting the channel [27]. Traditionally, channel modeling employs Fick’s second law to describe diffusion processes. However, to account for fluid dynamic effects, this study adopts the advection–diffusion equation (as detailed in Section 3) for a more comprehensive characterization of molecular propagation within the channel.

## 3. Channel Modeling

In MC systems, the transmitter releases information-carrying molecules at a fixed initial velocity. During propagation, these molecules are influenced by Brownian motion and fluid dynamics. Traditional channel models typically assume that molecules propagate under free-diffusion conditions. However, in practical applications, the viscous force and convective effects of the fluid have a significant impact on molecular motion. To make the subsequent discussion of the SIMO model more accurate, it is necessary to model these influencing factors in the SISO model and derive the expression for molecular concentration, which will then be extended to the SIMO model.

For the SIMO model, the position of the receiver in three-dimensional space affects the number of molecules it receives, such as the occlusion effects between different receivers, the distance, and the angle between the receiver and the transmitter [24]. These factors influence the spatial distribution of molecular concentration, thereby affecting the reception levels of different receivers.

**Figure 4:**
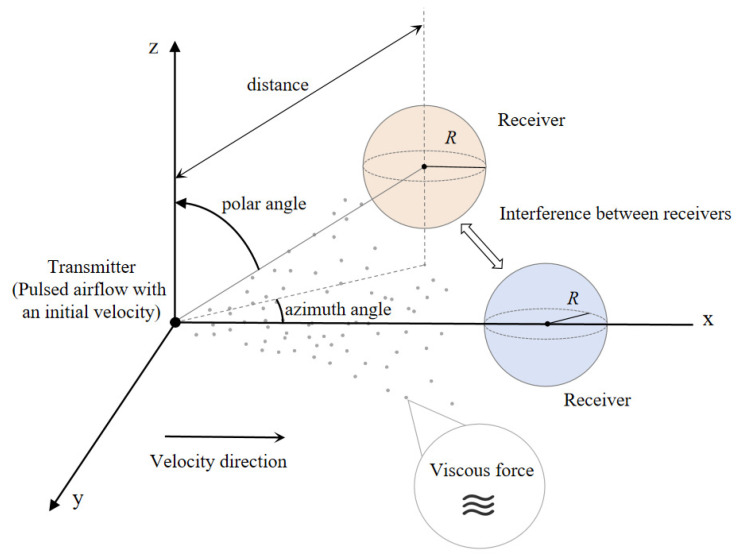
The three-dimensional spatial model of a SIMO molecular communication system. The transmitter releases molecular signals through pulsed airflow with an initial velocity, and the molecules diffuse in the propagation medium along the velocity direction while being influenced by viscous force. The receiver consists of two or more receptors located at different spatial positions. Spatial parameters such as polar angle, azimuth angle, and the distance between the transmitter and the receivers, as well as signal interference between the receivers, significantly affect the performance of the receivers.

In this section, we will delve into the derivation of formulas relevant to this study. The overall framework is centered around the convection–diffusion model, aiming to analyze the propagation process of messenger molecules from the transmitter (Tx) to the receiver (Rx). The structure of the framework is illustrated in Figure 4. We first consider the effects of convection and diffusion on the propagation of molecules within the channel. After being released from the Tx, the molecules diffuse through the channel and are subjected to resistance from the surrounding fluid during their propagation. This resistance arises from the viscous force between the channel medium and the molecules, which influence the propagation distance and velocity of the molecules [23].

In our SIMO model, the position of the Rx has a significant impact on the reception probability of molecules, such as the occlusion effects between different receivers, as well as the distance and angle between the receiver and the transmitter [24]. These factors not only influence the concentration distribution of viral molecules but are also directly related to the number of molecules that the receiver can capture. During the derivation process, we comprehensively considered the aforementioned factors; mathematically modeled the impulsive role of impulsive force in the model, the effects of viscous force, and the interference among multiple receivers; and provided an approximate concentration expression for the SIMO MC system.

### 3.1. Channel Impulse Response for Pulse-Assisted SISO MC System

In the SISO MC system, the transmitter releases molecules at a fixed initial velocity. During propagation, these molecules are influenced by Brownian motion and fluid dynamics. Traditional channel models typically assume that molecules propagate under free-diffusion conditions. However, in practical applications, the viscous force and convection effects of the fluid have a significant impact on the movement of the molecules. To make the subsequent discussion of the SIMO model more accurate, it is therefore necessary to model these influencing factors in the SISO model and derive the expression for molecular concentration, which will then be extended to the SIMO model.

**Figure 5:**
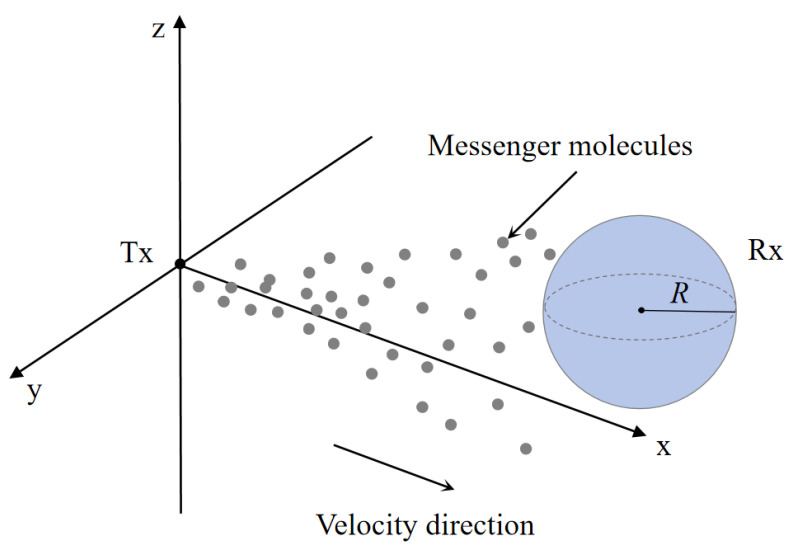
A three-dimensional spatial SISO molecular communication system model based on impulsive pulses.

The channel model of a SISO MC system assisted by impulsive force will be analyzed below, as shown in Figure 5. This scenario is set in an unbounded three-dimensional space, primarily deriving the instantaneous concentration of molecules in the space. It is assumed that the Tx is an infinitesimally small point emitter located at the center of the three-dimensional medium. It instantaneously releases molecules with an initial velocity of $u_{0}$, with the velocity direction parallel to the positive x-axis. The propagation of the molecules is influenced by Brownian motion and medium resistance, and the diffusion coefficient D of the molecules remains constant in the environment. The Rx is distributed in the positive x-axis region of the three-dimensional space, i.e., x>0. We assume that Rx is a spherical receiver with a radius of *R*, and when molecules come into contact with Rx, they are absorbed and removed from the environment.

According to the law of conservation of mass, the following equations can be derived [28,29]:

(1) $$\frac{\partial C(x,y,z,t)}{\partial t}+\nabla \cdot \vec{F}=S\left(x,y,z,t\right),$$

where $\frac{\partial C(x,y,z,t)}{\partial t}$ represents the change in molecular concentration over time, S(x,y,z,t) is the molecular source term released by the emitter at the initial moment, and $\vec{F}$ is the mass flux, which includes the diffusion component ${\vec{F}}_{diff}$ and the convection component ${\vec{F}}_{adv}$. There are many methods to derive $\vec{F}$, which largely depend on the environment. We focus on extending this term to include Fick’s convection and diffusion effects [13], expressed as

(2) $$\vec{F}={\vec{F}}_{\mathrm{diff}}+{\vec{F}}_{\mathrm{adv}}.$$

For convective flux, the overall transport of particles is caused by the motion of the fluid within the space. In our scenario, the convective velocity exists only along direction x, while in directions y and z, only the molecular diffusion flux is present. The vector velocity can be expressed as $\vec{v}=\left[u(t),0,0\right]$, where u(t) represents the molecular velocity equation. Therefore, the convective flux ${\vec{F}}_{\mathrm{adv}}$ is given by

(3) $${\vec{F}}_{\mathrm{adv}}=\vec{v}C\left(x,y,z,t\right).$$

Due to the incompressible nature of the fluid, its density remains constant, which implies $\nabla \cdot \vec{v}=0$ [13]. Consequently, the divergence of ${\vec{F}}_{\mathrm{adv}}$ can be derived as

(4) $$\nabla \cdot {\vec{F}}_{\mathrm{adv}}=\vec{v}\left(\nabla C\left(x,y,z,t\right)\right)=u\left(t\right)\frac{\partial C(x,y,z,t)}{\partial x}$$

According to Fick’s law of diffusion, the flux caused by diffusion is proportional to the concentration gradient:

(5) $${\vec{F}}_{\mathrm{diff}}=-D\nabla C\left(x,y,z,t\right),$$

where *D* represents the diffusion coefficient, which reflects the speed of molecular diffusion within a medium and is typically determined by the surrounding medium. Due to the thermal motion of molecules, diffusion occurs in all three directions—x, y, and z—resulting in diffusion fluxes. Therefore, the divergence of the diffusion component ${\vec{F}}_{\mathrm{diff}}$ can be expressed as

(6) $$\begin{array}{c} \nabla \;\cdot \;{\vec{F}}_{\mathrm{diff}}=D\left(\frac{\partial^{2}C\left(x,y,z,t\right)}{\partial x^{2}}+\frac{\partial^{2}C\left(x,y,z,t\right)}{\partial y^{2}}+\frac{\partial^{2}C\left(x,y,z,t\right)}{\partial z^{2}}\right). \end{array}$$

To derive a closed-form expression, certain boundary conditions need to be considered: C(∞,y,z,t)=0, C(x,∞,z,t)=0, and C(x,y,∞,t)=0. Additionally, the direction of the diffusion flux is opposite to that of the concentration gradient. Based on Equations (1), (4), and (6), the final convective–diffusion equation can be obtained:

(7) $$\begin{array}{cc} \; & \frac{\partial C(x,y,z,t)}{\partial t}+u\left(t\right)\frac{\partial C(x,y,z,t)}{\partial x}-D(\frac{\partial^{2}C\left(x,y,z,t\right)}{\partial x^{2}}\\ \; & +\frac{\partial^{2}C\left(x,y,z,t\right)}{\partial y^{2}}+\frac{\partial^{2}C\left(x,y,z,t\right)}{\partial z^{2}})=S\left(x,y,z,t\right). \end{array}$$

We primarily study pulse-assisted MC propagation modeling to understand its dynamic characteristics in the time and space domains. At the initial time t=0, Tx emits *N* molecules at position (x=0,y=0,z=0) as the source term of the system, leading to the following differential equation:

(8) $$\begin{array}{cc} \; & \frac{\partial C(x,y,z,t)}{\partial t}+u\left(t\right)\frac{\partial C(x,y,z,t)}{\partial x}-D(\frac{\partial^{2}C\left(x,y,z,t\right)}{\partial x^{2}}\\ \; & +\frac{\partial^{2}C\left(x,y,z,t\right)}{\partial y^{2}}+\frac{\partial^{2}C\left(x,y,z,t\right)}{\partial z^{2}})=N\delta \left(x\right)\delta \left(y\right)\delta \left(z\right)\delta \left(t\right). \end{array}$$

Moreover, it is necessary to consider the effect of pulse assistance on molecular displacement along direction x. Let the resulting displacement be denoted as s(t), which is related to u(t) as

(9) s(t)=∫u(t)dt.

Based on computational experience, we can derive the concentration expression for this scenario from the above differential equation using the Laplace transform [13]. The specific steps can be found in Appendix A.1:

(10) $$C\left(x,y,z,t\right)=\frac{N}{{\left(4\pi Dt\right)}^{\frac{3}{2}}}\exp\left(-\frac{{\left(x-s(t)\right)}^{2}+y^{2}+z^{2}}{4Dt}\right).$$

Next, we will delve into a detailed discussion of the impact of viscous force on the propagation of molecules in a fluid. After being emitted by the Tx, molecules are subjected to the resistance of the surrounding fluid, which is primarily caused by viscous force [23]. Viscous force arises from the relative motion between the molecules and the fluid and is typically described by Stokes’ law. Stokes’ law states that the resistance exerted by the fluid on a moving object is proportional to the object’s velocity, with the specific expression given as

(11) F(t)=−6πηru(t),

where η represents the viscosity of the fluid, and *r* represents the radius of the molecule. The direction of F(t) is opposite to the direction of the molecule’s motion. Therefore, molecules tend to decelerate during motion. The force situation can be expressed according to Newton’s second law:

(12) $$F\left(t\right)=m\frac{du(t)}{dt},$$

where *m* is the molecular mass, and substituting the viscous force into Newton’s second law, it follows from Equations (11) and (12) that

(13) $$-6\pi \eta ru\left(t\right)=m\frac{du(t)}{dt}.$$

Separating the variables of the equation with respect to u(t) and *t*, we obtain the following:

(14) $$\frac{du(t)}{u(t)}=-\frac{6\pi \eta r}{m}dt.$$

Integrating both sides, since u(t) always points in the positive x-direction, Equation (14) becomes

(15) $$\ln\left(u(t)\right)=-\frac{6\pi \eta r}{m}t+M,$$

where *M* is the constant. At t=0, the velocity $u\left(0\right)=u_{0}$. Substituting the initial condition, we obtain $M=\ln u_{0}$. Taking the exponential on both sides of Equation (15), the velocity formula for the numerator u(t) is obtained as

(16) $$u\left(t\right)=u_{0}\exp\left(-\frac{6\pi \eta rt}{m}\right).$$

Therefore, the displacement s(t) can be obtained by integrating u(t) with respect to time *t*.

(17) $$S\left(t\right)=\int u\left(t\right)dt=\int u_{0}\exp\left(-\frac{6\pi \eta rt}{m}\right)dt.$$

Using the method of variable substitution to solve the integral equation, let $\tau =-\frac{6\pi \eta rt}{m}$, then $\frac{d\tau }{dt}=-\frac{6\pi \eta r}{m}$, which means $dt=-\frac{m}{6\pi \eta r}d\tau$. Substituting into the integral yields

(18) $$s\left(t\right)=-\frac{u_{0}m}{6\pi \eta r}\exp\left(-\frac{6\pi \eta rt}{m}\right)+M^{\prime }.$$

Then, solve for the constant $M^{\prime }$. Since at t=0, the particle has not been emitted and is located at the origin, set the initial condition as displacement s(0)=0. Thus, we obtain

(19) $$s\left(0\right)=-\frac{u_{0}m}{6\pi \eta r}\exp\left(0\right)+M^{\prime }=0.$$

The determined integral constant can thus be solved as

(20) $$M^{\prime }=\frac{u_{0}m}{6\pi \eta r}.$$

Substitute Equation (20) into Equation (18) to obtain the complete displacement equation:

(21) $$s\left(t\right)=\frac{u_{0}m}{6\pi \eta r}\left(1-\exp\left(-\frac{6\pi \eta rt}{m}\right)\right).$$

Based on Equations (8) and (21), the molecular concentration expression considering the effects of fluid velocity and viscous force can be derived as follows:

(22) $$\begin{array}{cc} \; & C\left(x,y,z,t\right)=\frac{N}{{\left(4\pi Dt\right)}^{\frac{3}{2}}}\exp\left(\frac{-{\left(x-\frac{u_{0}m}{6\pi \eta r}\left(1-\exp\left(-\frac{6\pi \eta rt}{m}\right)\right)\right)}^{2}-y^{2}-z^{2}}{4Dt}\right). \end{array}$$

Based on the molecular concentration expression (22) of the SISO model, we integrate this formula within the spherical receiver Rx’s range to obtain the total number of molecules instantaneously received within the Rx range:

(23) $$\begin{array}{c} Q\left(\mathrm{Rx},t\right)={\;\int \int \int \;}_{V_{\mathrm{Rx}}}C\left(x,y,z,t\right)\;dV={\;\int \int \int \;}_{V_{\mathrm{Rx}}}\frac{N}{{\left(4\pi Dt\right)}^{\frac{3}{2}}}\exp\;\left(\frac{-{\left(x\;-\;\frac{u_{0}m}{6\pi \eta r}\;\left(1\;-\;\exp\;\left(-\frac{6\pi \eta rt}{m}\right)\right)\right)}^{2}-y^{2}\;-\;z^{2}}{4Dt}\right)dV. \end{array}$$

Through the above derivation, we obtained the concentration expression for the SISO MC system under pulse assistance, analyzed the impact of viscous force on molecular propagation, and determined the instantaneous total number of molecules received by the Rx in space. This result provides a foundation for the subsequent extension to the SIMO model.

### 3.2. Channel Impulse Response for SIMO MC System

In this section, we will explore the expression for the number of molecules received in a pulse-assisted SIMO MC system and consider the interference effects among multiple receivers.

In the SIMO system, multiple Rx coexist within the channel, and these receivers may interfere with each other, leading to a reduction in the number of molecules received by the primary receiver (i.e., the target receiver). Specifically, when the Tx releases messenger molecules, these molecules undergo diffusive motion in the medium and are absorbed by the receivers. However, due to the relative positions and orientations of the receivers, some molecules may be absorbed by other receivers before reaching the target receiver, thereby affecting the reception efficiency of the primary receiver. Therefore, understanding and modeling this interference effect is key to optimizing the performance of MC systems.

To address the aforementioned issue, we will establish a mathematical model to describe the number of molecules received by the primary receiver in the presence of multiple receivers. We will first derive the expression for the number of molecules received by a single receiver in three-dimensional space, then quantify the interference effects of other receivers on the primary receiver, and finally obtain an approximate expression for the number of molecules received by the primary receiver in a multi-receiver environment.

**Figure 6:**
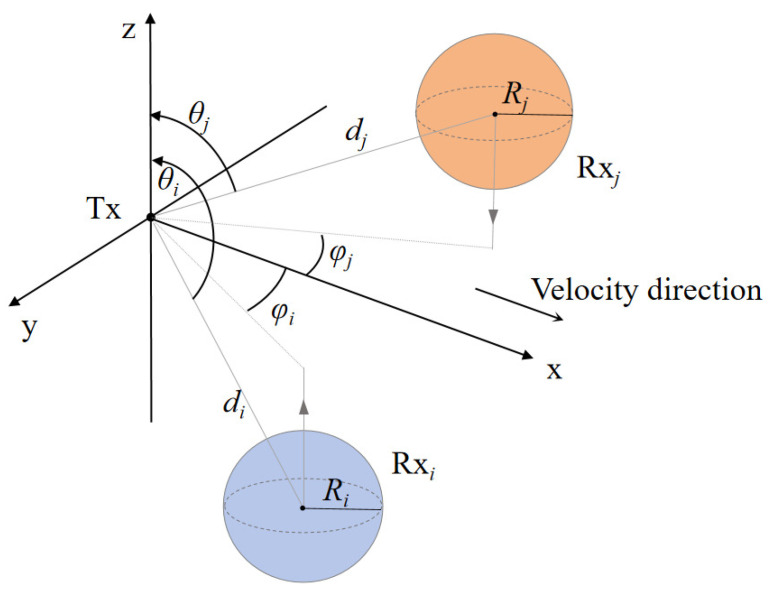
A pulse-assisted SIMO MC system model with a Tx and two spherical absorbing receivers $Rx_{i}$ and $Rx_{j}$, along with the associated spatial parameters.

First, we consider a model consisting of a Tx and *n* spherical Rx located at different positions in three-dimensional space, as shown in Figure 6. The radius of each receiver $Rx_{i}$ is $R_{i}$, and the shortest distance between its center and the transmitter Tx is *d*, with a polar angle of θ(0≤θ≤π) and an azimuthal angle of $\varphi \;(-\frac{\pi }{2}\leq \varphi \leq \frac{\pi }{2})$.

To facilitate the study of factors such as distance, angle, and interference between multiple receivers, it is first necessary to convert Equation (23) from the Cartesian coordinate system to the spherical coordinate system. The spherical coordinates of $Rx_{i}$ are denoted as $\left(d_{i},\theta_{i},\varphi_{i}\right)$, based on the following formula:

(24) $$\begin{array}{cc} x & =d_{i}\sin\theta_{i}\cos\varphi_{i}+d\sin\theta \cos\varphi ,\\ y & =d_{i}\sin\theta_{i}\sin\varphi_{i}+d\sin\theta \sin\varphi ,\\ z & =d_{i}\cos\theta_{i}+d\cos\theta . \end{array}$$

The expression for the number of receptor molecules in the three-dimensional space of $Rx_{i}$ can be obtained as Equation (25). We use the trigonometric identity $\sin^{2}A\sin^{2}B+\cos^{2}A\sin^{2}B+\cos^{2}B=1$ and the cosine difference formula cos(A−B)=cosAcosB+sinAsinB. This allows us to simplify it to Equation (26).

(25) $$\begin{array}{cc} \; & Q^{\mathrm{SISO}}\left(Rx_{i},t\right)={\;\int \int \int \;}_{V_{Rx_{i}}}C\left(d,\theta ,\varphi ,t\right)dV\\ \; & ={\;\int \int \int \;}_{V_{Rx_{i}}}\frac{N}{{\left(4\pi Dt\right)}^{\frac{3}{2}}}\exp\left(-\frac{{\left(d_{i}\sin\theta_{i}\cos\varphi_{i}+d\sin\theta \cos\varphi -\frac{u_{0}m}{6\pi \eta r}\left(1-\exp\left(-\frac{6\pi \eta rt}{m}\right)\right)\right)}^{2}}{4Dt}\right)\\ \; & \times \exp\left(\frac{-{\left(d_{i}\sin\theta_{i}\sin\varphi_{i}-d\sin\theta \sin\varphi \right)}^{2}+{\left(d_{i}\cos\theta_{i}+d\cos\theta \right)}^{2}}{4Dt}\right)d^{2}\sin\theta ddd\theta d\varphi \\ \; & =\int_{0}^{2\pi }\int_{0}^{\pi }\int_{0}^{R_{i}}\frac{N}{{\left(4\pi Dt\right)}^{\frac{3}{2}}}\exp\left(-\frac{{\left(d_{i}\sin\theta_{i}\cos\varphi_{i}+d\sin\theta \cos\varphi -\frac{u_{0}m}{6\pi \eta r}\left(1-\exp\left(-\frac{6\pi \eta rt}{m}\right)\right)\right)}^{2}}{4Dt}\right)d^{2}\sin\theta ddd\theta d\varphi \\ \; & \times \exp\left(\frac{-{\left(d_{i}\sin\theta_{i}\sin\varphi_{i}-d\sin\theta \sin\varphi \right)}^{2}+{\left(d_{i}\cos\theta_{i}+d\cos\theta \right)}^{2}}{4Dt}\right)d^{2}\sin\theta ddd\theta d\varphi . \end{array}$$

(26) $$\begin{array}{cc} \; & Q^{\mathrm{SISO}}\left(Rx_{i},t\right)=\int_{0}^{2\pi }\int_{0}^{\pi }\int_{0}^{R_{i}}\frac{Nd^{2}\sin\theta }{{\left(4\pi Dt\right)}^{\frac{3}{2}}}\exp\left(-\frac{d^{2}+{d_{i}}^{2}-\frac{u_{0}m\left(d_{i}\sin\theta_{i}\cos\varphi_{i}+d\sin\theta \cos\varphi \right)}{3\pi \eta r}\left(1-\exp\left(-\frac{6\pi \eta rt}{m}\right)\right)}{4Dt}\right)\\ \; & \times \exp\left(-\frac{{\left(\frac{u_{0}m}{6\pi \eta r}\left(1-\exp\left(-\frac{6\pi \eta rt}{m}\right)\right)\right)}^{2}+2dd_{i}\left(\sin\theta_{i}\sin\theta \cos\left(\varphi -\varphi_{i}\right)+\cos\theta_{i}\cos\theta \right)}{4Dt}\right)ddd\theta d\varphi . \end{array}$$

**Figure 7:**
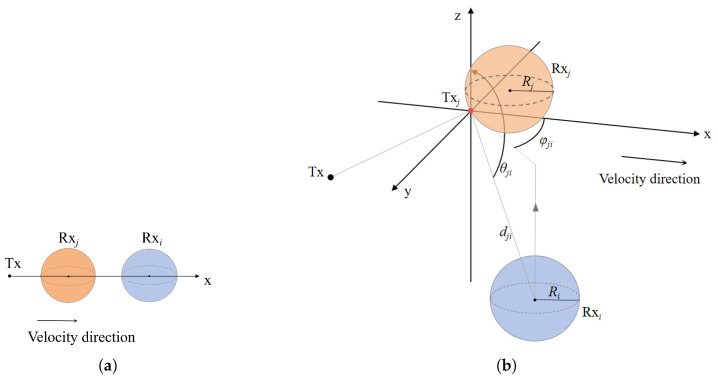
(**a**) The scenario where $Rx_{j}$ occludes $Rx_{i}$. (**b**) A schematic model illustrating the influence of the interfering receiver $Rx_{j}$ on the primary receiver $Rx_{i}$. Molecules emitted by Tx, which were originally intended to be absorbed by $Rx_{i}$, are instead absorbed by the absorption point $Tx_{j}$ of $Rx_{j}$.

The main difference between SIMO systems and SISO systems lies in the fact that the former needs to handle interference among different Rx values. In the same environment, the molecular reception of a single Rx in a SIMO system is lower than that in a SISO system, i.e., $Q^{\mathrm{SIMO}}\left(Rx_{i},t\right)\leq Q^{\mathrm{SISO}}\left(Rx_{i},t\right)$, which is precisely due to the interference caused by other Rx. In SIMO, molecular absorption competition is an important phenomenon that affects the quality of signal transmission. Certain molecules may be absorbed by other receivers before reaching the target receiver, thereby reducing the number of molecules received by the target receiver. This effect is mutual, as each receiver interferes with the spatial domain relative to the other receivers. If $Rx_{j}$ is positioned ahead of $Rx_{i}$ in the x-direction, the molecules that would have originally been absorbed by $Rx_{i}$ will instead be absorbed by $Rx_{j}$, leading to a reduction in the amount received, as shown in Figure 7a. Due to the independence of motion increments, the absorption rate of the molecules absorbed by $Rx_{j}$ can be modeled independently, as if they were not absorbed by $Rx_{j}$ but rather released from the absorption point of $Rx_{j}$.

When molecules propagate, they generally collide with the front side of the Rx facing the Tx, as shown in Figure 7b. We define the point on the surface of $Rx_{i}$ closest to Tx as approximately the center of the absorbed molecule, denoted as $Tx_{j}$ [30]. Based on this approximation, the distance from the center of the molecule absorbed by $Rx_{j}$ to the center of the sphere $Rx_{i}$ is represented as $d_{ji}$. The polar angle between the center of the molecule absorbed by $Rx_{i}$ and $Rx_{i}$ itself is denoted as $\theta_{ji}$, while the azimuthal angle between the center of the molecule absorbed by $Rx_{i}$ and $Rx_{i}$ is denoted as $\varphi_{ji}$. Thus, the spherical coordinates of $Rx_{i}$ relative to $Tx_{j}$ are represented as $\left(d_{ji},\theta_{ji},\varphi_{ji}\right)$.

From a comprehensive perspective, the molecules ultimately absorbed by $Rx_{j}$ can be categorized into two sources: one originates from the release at Tx, and the other from the release at the absorption molecule center $Tx_{j}$ on $Rx_{j}$, as if they had not been absorbed. The latter accounts for the interference effect of $Rx_{j}$ on $Rx_{i}$. For this part, $Tx_{j}$ can be treated as a virtual release point, as if the molecules were released from that point. For example, molecules absorbed by $Rx_{j}$ at time τ<t can be regarded as having been released from the virtual release point $Tx_{j}$ at time τ [20].

In a pulse-assisted SIMO system, the number of molecules received by the main receiver Rx at time *t* should be expressed as the molecular concentration received by $Rx_{i}$ in a SISO scenario, minus all the molecules stolen by Rx from $Rx_{i}$ before time *t*. The total amount of molecules stolen before time *t* is calculated by integrating over τ. The approximate model for the number of molecules received by the main receiver $Rx_{i}$ is given as

(27) $$\begin{array}{c} Q^{\mathrm{SIMO}}\left(Rx_{i},t\right)\approx Q^{\mathrm{SISO}}\left(Rx_{i},t\right)-\sum_{\begin{array}{c} j=1\\ j\neq i \end{array}}^{n}\int_{0}^{t}Q^{\mathrm{SIMO}}\left(Rx_{j},\tau \right)Q^{\mathrm{SISO}}\left(Rx_{i},t|Rx_{j},\tau \right)d\tau ,\left(t>\tau \right). \end{array}$$

where $Q^{\mathrm{SISO}}\left(Rx_{i},t\right)$ represents the number of molecules received by $Rx_{i}$ at time *t* in the absence of interference from other receivers; $Q^{\mathrm{SIMO}}\left(Rx_{j},\tau \right)$ represents the number of molecules received by $Rx_{j}$ at time τ in the SIMO scenario; and $Q^{\mathrm{SISO}}\left(Rx_{i},t|Rx_{j},\tau \right)$ refers to the number of molecules that were originally intended to reach $Rx_{i}$ but were intercepted by $Rx_{j}$ at time τ prior to time *t*. The specific expression for this can be found in Equation (28):

(28) $$\begin{array}{cc} \; & Q^{\mathrm{SISO}}\left(Rx_{i},t|Rx_{j},\tau \right)=\int_{0}^{2\pi }\int_{0}^{\pi }\int_{0}^{R_{j}}\frac{Nd^{2}\sin\theta }{{\left(4\pi D\tau \right)}^{\frac{3}{2}}}\exp\left(-\frac{d^{2}+{d_{ji}}^{2}-\frac{u_{0}m\left(d_{i}\sin\theta_{i}\cos\varphi_{i}+d\sin\theta \cos\varphi \right)}{3\pi \eta r}\left(1-\exp\left(-\frac{6\pi \eta r\tau }{m}\right)\right)}{4D\tau }\right)\\ \; & \times \exp\left(-\frac{{\left(\frac{u_{0}m}{6\pi \eta r}\left(1-\exp\left(-\frac{6\pi \eta r\tau }{m}\right)\right)\right)}^{2}+2dd_{ji}\left(\sin\theta_{ji}\sin\theta \cos\left(\varphi -\varphi_{ji}\right)+\cos\theta_{ji}\cos\theta \right)}{4D\tau }\right)ddd\theta d\varphi . \end{array}$$

Since this term only represents the number of molecules stolen by $Rx_{j}$ at moment τ, in a SIMO system with more than two receivers, it is necessary to sum them up. Additionally, integration over the time period from 0 to *t* is required to calculate the number of molecules stolen by other interfering receivers from $Rx_{i}$ at each moment. Furthermore, for the terms $d_{ji}$, $\theta_{ji}$, and $\varphi_{ji}$ included in the formula, their specific forms need to be determined. According to the law of cosines, the distance $d_{ji}$ from the molecular center absorbed by $Rx_{j}$ to $Rx_{i}$ can be expressed as Equation (29):

(29) $$d_{ji}=\sqrt{{\left(d_{j}-R_{j}\right)}^{2}+{d_{i}}^{2}-2\left(d_{j}-R_{j}\right)d_{i}\sin\theta_{i}\sin\theta_{j}\cos\left(\varphi_{i}-\varphi_{j}\right)+\cos\theta_{i}\cos\theta_{j}}.$$

From the polar coordinate angle formula, the polar angle $\theta_{ji}$ between the absorption molecular center $Tx_{j}$ of $Rx_{j}$ and $Rx_{i}$ is

(30) $$\theta_{ji}=\arccos\left(\frac{d_{i}\cos\theta_{i}-\left(d_{j}-R_{j}\cos\theta_{j}\right)}{d_{ji}}\right).$$

According to the law of cosines, the azimuth angle $\varphi_{ji}$ between $Tx_{j}$ and $Rx_{i}$ is

(31) $$\varphi_{ji}=\pi -\varphi_{j}-\arccos\left(\frac{{d_{ji}}^{2}+{\left(d_{j}-R_{j}\right)}^{2}-{d_{i}}^{2}}{2d_{ji}\left(d_{j}-R_{j}\right)}\right).$$

The expression for the number of molecules received by $Rx_{i}$ at time *t* in the final SIMO scenario is given as Equation (32):

(32) $$\begin{array}{cc} \; & Q^{\mathrm{SIMO}}\left(Rx_{i},t\right)\approx \int_{0}^{2\pi }\int_{0}^{\pi }\int_{0}^{R_{i}}\frac{Nd^{2}\sin\theta }{{\left(4\pi Dt\right)}^{3/2}}\exp\left(-\frac{d^{2}+d_{i}^{2}-\frac{u_{0}m\left(d_{i}\sin\theta_{i}\cos\phi_{i}+d\sin\theta \cos\phi \right)}{3\pi \eta r}\left(1-\exp\left(-\frac{6\pi \eta r\tau }{m}\right)\right)}{4Dt}\right)\\ \; & \times \exp\left(-\frac{{\left(\frac{u_{0}m}{6\pi \eta r}\left(1-\exp\left(-\frac{6\pi \eta r\tau }{m}\right)\right)\right)}^{2}+2dd_{i}\left(\sin\theta_{i}\sin\theta \cos\left(\phi -\phi_{i}\right)+\cos\theta_{i}\cos\theta \right)}{4Dt}\right)dd\;d\theta \;d\varphi \\ \; & -\sum_{j=1,j\neq i}^{n}\frac{N^{2}}{{\left(4\pi Dt\right)}^{3}}\int_{0}^{t}(\int_{0}^{2\pi }\int_{0}^{\pi }\int_{0}^{R_{j}}d^{2}\sin\theta \exp\left(-\frac{d^{2}+d_{j}^{2}-\frac{u_{0}m\left(d_{j}\sin\theta_{j}\cos\phi_{j}+d\sin\theta \cos\phi \right)}{3\pi \eta r}\left(1-\exp\left(-\frac{6\pi \eta r\tau }{m}\right)\right)}{4Dt}\right)\\ \; & \times \exp\left(-\frac{{\left(\frac{u_{0}m}{6\pi \eta r}\left(1-\exp\left(-\frac{6\pi \eta r\tau }{m}\right)\right)\right)}^{2}+2dd_{j}\left(\sin\theta_{j}\sin\theta \cos\left(\phi -\phi_{j}\right)+\cos\theta_{j}\cos\theta \right)}{4Dt}\right)\;dd\;d\theta \;d\varphi \\ \; & \times \int_{0}^{2\pi }\int_{0}^{\pi }\int_{0}^{R_{i}}d^{2}\sin\theta \exp\left(-\frac{d^{2}+d_{ji}^{2}-\frac{u_{0}m\left(d_{ji}\sin\theta_{ji}\cos\phi_{ji}+d\sin\theta \cos\phi \right)}{3\pi \eta r}\left(1-\exp\left(-\frac{6\pi \eta r\tau }{m}\right)\right)}{4Dt}\right)\\ \; & \times \exp\left(-\frac{{\left(\frac{u_{0}m}{6\pi \eta r}\left(1-\exp\left(-\frac{6\pi \eta r\tau }{m}\right)\right)\right)}^{2}+2dd_{ji}\left(\sin\theta_{ji}\sin\theta \cos\left(\phi -\phi_{ji}\right)+\cos\theta_{ji}\cos\theta \right)}{4Dt}\right)\;dd\;d\theta \;d\varphi \;)d\tau ,\;\left(t>\tau \right). \end{array}$$

## 4. Results

### 4.1. Simulation Settings

In this section, we conducted simulation experiments using MATLAB R2023b. Unless otherwise specified, all experiments followed the conditions outlined in Table 2. The experimental scenario includes a point Tx and several spherical absorbing Rx. Each Rx has a radius *R* of $2\times 10^{-3}$ m. Each scenario in the experiment includes at least one Tx and one primary Rx, with some scenarios also incorporating interfering Rx to study their impact on the primary Rx. The Tx is located at the origin, and each Rx is positioned within the positive x-direction range in the space. Multiple simulations were conducted by varying parameters such as *d*, θ, and φ of the Rx in the scenarios, along with other factors.

In the simulation experiments, we employed the Monte Carlo method to randomly simulate molecular motion, with a time step set to $10^{-3}$ s. During each time step, the concentration distribution of molecules in space is updated. To reduce the impact of random errors on the results, all experiments were conducted independently, totaling 100 repetitions. The results of each experiment were obtained by calculating the average number of molecules received by the Rx over a specific time period. To ensure convergence, the relative standard deviation of the experimental results was set to be less than 5%. In terms of numerical integration, we used the finite difference method to solve the derived theoretical formulas. To validate the effectiveness of the model, we also compared the simulation data with the theoretical model data.

First, we will consider the impact of the angles θ and φ, as well as the distance *d*, on the number of molecules received by the Rx in the SISO model. The angles θ and φ represent the angular separation between the receiver Rx and the positive x-direction (i.e., the direction of the impulse force). The distance *d* reflects the proximity between the receiver Rx and the transmitter Tx. These factors collectively illustrate how the position of Rx in three-dimensional space influences the number of molecules it receives. Variations in these parameters lead to different reception responses. Subsequently, in the SIMO model, the effect of interfering Rx positions on the primary Rx is analyzed. The following content examines several scenarios designed based on the positions of the receivers.

### 4.2. The Angle Influences the Number of Molecules Received

**Figure 8:**
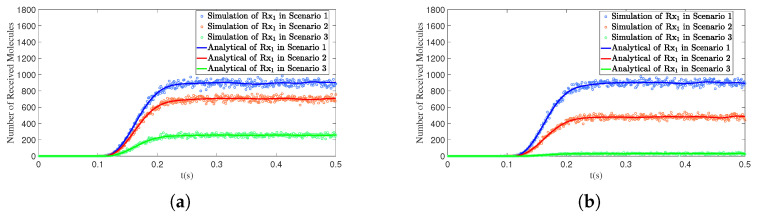
By comparing simulation and model results, the variation in the number of molecules received by the receivers over time *t* is obtained under different polar angles θ and azimuth angles ϕ: (**a**) Scenario 1. $d_{1}=1.00\;m,\;\theta_{1}=90^{\circ },\;\phi_{1}=0^{\circ }$; Scenario 2. $d_{2}=1.00\;m,\;\theta_{2}=80^{\circ },\;\phi_{2}=0^{\circ }$; Scenario 3. $d_{3}=1.00\;m,\;\theta_{3}=70^{\circ },\;\phi_{3}=0^{\circ }.$ (**b**) Scenario 1. $d_{1}=1.00\;m,\;\theta_{1}=90^{\circ },\;\phi_{1}=0^{\circ }$; Scenario 2. $d_{2}=1.00\;m,\;\theta_{2}=90^{\circ },\;\phi_{2}=15^{\circ }$; Scenario 3. $d_{3}=1.00\;m,\;\theta_{3}=90^{\circ },\;\phi_{3}=30^{\circ }$.

This section primarily discusses the impact of two angles on the number of received molecules in the SISO model. In Figure 8a,b, three experiments were conducted for both the polar angle θ and the azimuthal angle φ. Specifically, in Figure 8a, the three Rx receivers varied in their polar angle θ, while in Figure 8b, the Rx receivers varied in their azimuthal angle φ. However, the results in both figures are similar, showing that increasing the separation angle leads to a reduction in the number of received molecules.

**Figure 9:**
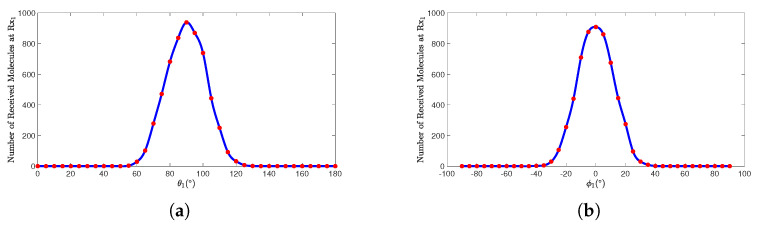
When $d_{1}=1.00\;m$ and t=1s, the number of molecules received by receiver ${\mathrm{Rx}}_{1}$ varies with (**a**) the polar angle $\theta_{1}$ and (**b**) the azimuth angle $\phi_{1}$.

Figure 9a,b, respectively, illustrate the graphs of the number of molecules received by $Rx_{1}$ in a SISO scenario as a function of the polar angle $\theta_{1}$ and azimuthal angle $\varphi_{1}$ of $Rx_{1}$. When $d_{1}$ and *t* remain constant, as the polar angle increases from $60^{\circ }$ to $120^{\circ }$ and the azimuth angle ranges from ${-30}^{\circ }$ to $30^{\circ }$, the curves exhibit a trend of first increasing and then decreasing. The peaks of the curves are located near $\varphi_{1}$ = $0^{\circ }$ and $\theta_{1}$ = $90^{\circ }$, and these curves display an axisymmetric distribution centered around $\varphi_{1}$ = $0^{\circ }$ and $\theta_{1}$ = $90^{\circ }$. Further analysis reveals that the reception efficiency is highest when the receiver is positioned directly in front of the transmitter’s pulse direction, while the efficiency decreases as the angle deviates further from the pulse direction.

The angular position of the receiver directly affects the effectiveness of the molecular propagation path. When the receiver is positioned directly in front of the transmitter, the directionality of molecular diffusion is strongest, resulting in the highest reception efficiency. However, as the angle deviates, the effective coverage range of molecular diffusion decreases, leading to a reduction in reception efficiency or even complete loss of reception. Therefore, in the design of pulse-assisted MC systems, it is crucial to strategically plan the receiver’s position and control the angular deviation within a certain range to enhance the reception efficiency of information molecules, thereby achieving better propagation performance.

### 4.3. The Distance Influences the Number of Molecules Received

**Figure 10:**
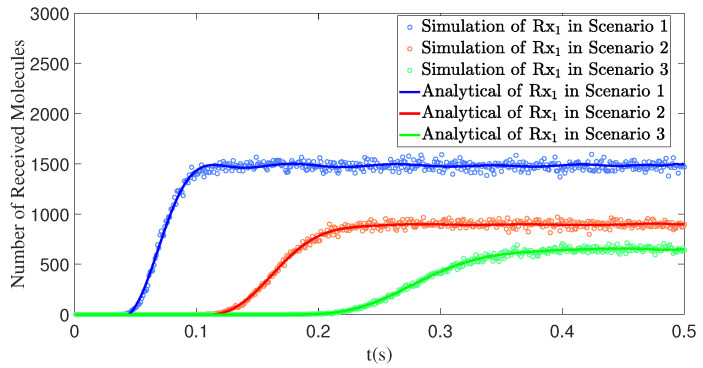
By comparing simulation and model results, the variation in the number of molecules received by the receivers over time *t* is obtained for different distances *d*. $Rx_{1}$: $d_{1}=0.50\;m,\;\theta_{1}=90^{\circ },\;\phi_{1}=0^{\circ }$; $Rx_{2}$: $d_{2}=1.00\;m,\;\theta_{2}=90^{\circ },\;\phi_{2}=0^{\circ }$; $Rx_{3}$: $d_{3}=1.50\;m,\;\theta_{3}=90^{\circ },\;\phi_{3}=0^{\circ }$.

This section primarily discusses the impact of the distance *d* between the Rx and the Tx, as well as the initial velocity $u_{0}$, on the number of received molecules in the SISO model. Figure 10 presents three experiments focusing on the distance *d*. Compared to $Rx_{2}$ and $Rx_{3}$, $Rx_{1}$ is closer to the Tx, which allows it to start receiving molecules earlier. Additionally, the stable range of the number of received molecules for $Rx_{1}$ is higher than that of the other two.

**Figure 11:**
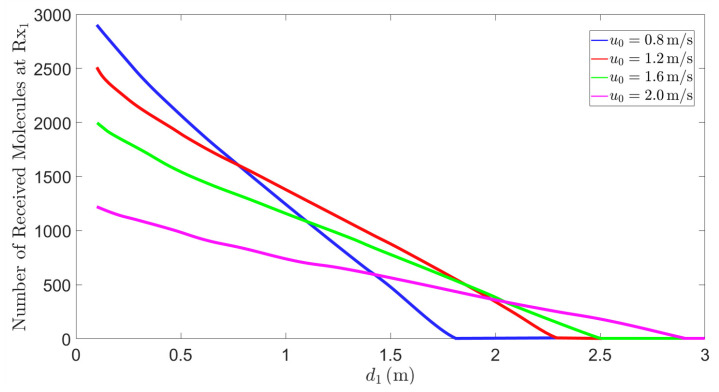
When t=1s, $\theta_{1}=90^{\circ }$, and $\phi_{1}=0^{\circ }$, the number of molecules received by $Rx_{1}$ varies with the distance $d_{1}$ under different initial velocities $u_{0}$. The distance $d_{1}$ ranges from 0.1m to 3m.

According to Figure 11, when the polar angle $\theta_{1}$ and the azimuthal angle $\varphi_{1}$ remain fixed, the curve exhibits a monotonically decreasing trend as $d_{1}$ increases. As the distance increases, the concentration of molecules within the propagation range gradually decreases due to the effects of viscous resistance and diffusion during the propagation process, resulting in a reduction in the number of molecules received by the receiver. At the same time, we also observe that the initial velocity has an impact on the number of molecules received by the receiver. The maximum propagation distance of the molecules primarily depends on the initial velocity. Increasing $u_{0}$ can extend the propagation distance; however, due to the law of mass conservation, this leads to a decrease in the number of molecules received. Therefore, increasing the initial velocity is highly effective in enhancing the propagation range, but it may cause the reception rate to decrease within a certain range.

The effective range of MC is significantly limited by distance, as the intensity of molecular signals gradually attenuates with increasing distance between the transmitter and the receiver. In multi-user MC applications, shortening the distance between the transmitter and the receiver, while avoiding mutual interference among receivers, can effectively enhance the performance of MC. For example, in fields such as environmental monitoring and smart healthcare, designing compact network structures to reduce signal attenuation is crucial. Additionally, the initial velocity of emitted molecules also affects the propagation distance and the number of molecules received. When designing MC systems, it is essential to fully consider the impact of distance and the initial velocity of molecules on channel response in order to optimize the position of receivers as well as the magnitude and direction of molecular flow velocity, thereby improving system performance.

### 4.4. The Interfering Receiver Influences the Number of Molecules Received by the Primary Receiver

This section primarily explores the impact of the angular position θ, azimuth angle φ, distance *d*, and the number of interfering Rx on the number of molecules received by the primary Rx in a SIMO model with interfering receivers. The discussion is conducted through a comparison of two scenarios for each parameter and an analysis of the continuous variation curves of the parameters with respect to the number of received molecules.

#### 4.4.1. The Distance Between the Interfering Receiver and the Transmitter Affects the Number of Molecules Received by the Primary Receiver

**Figure 12:**
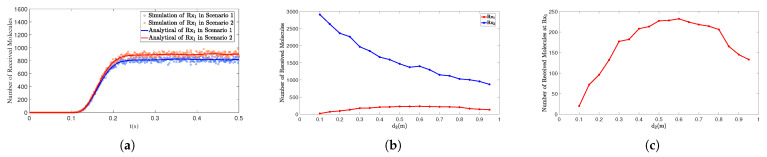
(**a**) By comparing simulation and model results, the number of molecules received by receiver $Rx_{1}$ over time *t* is obtained under different $d_{2}$. Scenario 1: $d_{1}=1.00\;m,\;\theta_{1}=90^{\circ },$ $\phi_{1}=0^{\circ };\;d_{2}=0.50\;m,\;\theta_{2}=70^{\circ },\;\phi_{2}=0^{\circ }$. Scenario 2: $d_{1}=1.00\;m,\;\theta_{1}=90^{\circ },\;\phi_{1}=0^{\circ };$ $d_{2}=1.00\;m,\;\theta_{2}=70^{\circ },\;\phi_{2}=0^{\circ }.$ (**b**) When t=1s, $d_{1}=1.00\;m$, $\theta_{1}=\theta_{2}=90^{\circ }$, and $\phi_{1}=\phi_{2}=0^{\circ }$, the number of molecules received by $Rx_{1}$ and $Rx_{2}$ varies with the distance $d_{2}$. The distance $d_{2}$ ranges from 0.1m to 0.95m. (**c**) The detailed variation of the number of molecules received by $Rx_{1}$ with the distance $d_{2}$.

Figure 12a illustrates the impact of the distance between the interfering receiver $Rx_{2}$ and the transmitter Tx on the number of molecules received by the primary receiver $Rx_{1}$ in two scenarios. In Scenario 1, $Rx_{2}$ is closer to Tx ($d_{2}$ = 0.50 m), resulting in a higher number of molecules received by $Rx_{2}$ and a stronger interference effect on $Rx_{1}$, which significantly reduces the number of molecules received by $Rx_{1}$. In Scenario 2, $Rx_{2}$ is farther from Tx ($d_{2}$ = 1.00 m), leading to a decrease in the number of molecules received by $Rx_{2}$ and a corresponding weakening of its interference effect on $Rx_{1}$, thereby increasing the number of molecules received by $Rx_{1}$.

Figure 12b,c further illustrate the impact of the continuous variation of $d_{2}$ on the number of molecules received by the primary receiver $Rx_{1}$ and the interfering receiver $Rx_{2}$. The position of the primary receiver $Rx_{1}$ is fixed, and as the distance $d_{2}$ between the interfering receiver $Rx_{2}$ and the transmitter Tx increases, the curves exhibit a trend of first increasing and then decreasing.

In Figure 12b, for $Rx_{2}$, as it is positioned in front of $Rx_{1}$ and is not affected by interference from the latter, the number of molecules it receives decreases monotonically with increasing distance. In Figure 12c, for $Rx_{1}$, as the distance between the interfering receiver $Rx_{2}$ and the transmitter Tx increases, the number of molecules received by the primary receiver $Rx_{1}$ first increases and then decreases. This is because when the interfering receiver is closer to either the transmitter or the primary receiver, its blocking effect on the primary receiver becomes more significant, thereby significantly reducing the number of molecules received by the primary receiver. When the interfering receiver is located at an intermediate position between the transmitter and the primary receiver, the molecular reception performance of the primary receiver reaches its optimum. In scenarios where receivers are densely arranged, such blocking effects may become more pronounced. By controlling the distance between $Rx_{2}$ and Tx, the interference experienced by $Rx_{1}$ can be minimized, thereby improving overall communication efficiency.

#### 4.4.2. The Angle Between the Interfering Receiver and the Transmitter Affects the Number of Molecules Received by the Primary Receiver

**Figure 13:**
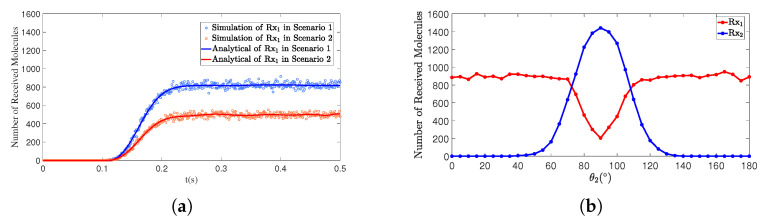
(**a**) By comparing simulation and model results, the number of molecules received by receiver $Rx_{1}$ over time *t* is obtained under different $\theta_{2}$. Scenario 1: $d_{1}=1.00\;m,\;\theta_{1}=90^{\circ },$ $\phi_{1}=0^{\circ };\;d_{2}=0.50\;m,\;\theta_{2}=70^{\circ },\;\phi_{2}=0^{\circ }$. Scenario 2: $d_{1}=1.00\;m,\;\theta_{1}=90^{\circ },\;\phi_{1}=0^{\circ };$ $d_{2}=0.50\;m,\;\theta_{2}=80^{\circ },\;\phi_{2}=0^{\circ }.$ (**b**) The polar angle $\theta_{2}$ of the interfering receiver $Rx_{2}$ varies from $0^{\circ }$ to $180^{\circ }$, while the distance to the transmitter $d_{2}$ is fixed at 0.50m, with an azimuth angle $\phi_{2}=0^{\circ }$. The convergence time is taken as t=1s.

**Figure 14:**
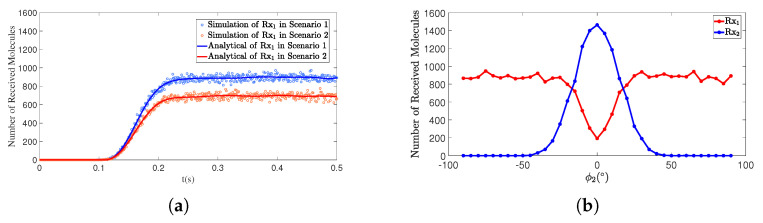
(**a**) By comparing simulation and model results, the number of molecules received by receiver $Rx_{1}$ over time *t* is obtained under different $\phi_{2}$. Scenario 1: $d_{1}=1.00\;m,\;\theta_{1}=90^{\circ },$ $\phi_{1}=0^{\circ };\;d_{2}=0.50\;m,\;\theta_{2}=90^{\circ },\;\phi_{2}=30^{\circ }$. Scenario 2: $d_{1}=1.00\;m,\;\theta_{1}=90^{\circ },\;\phi_{1}=0^{\circ };$ $d_{2}=0.50\;m,\;\theta_{2}=90^{\circ },\;\phi_{2}=15^{\circ }.$ (**b**) The azimuth angle $\phi_{2}$ of the interfering receiver $Rx_{2}$ varies from $-90^{\circ }$ to $+90^{\circ }$, while the distance to the transmitter $d_{2}$ is fixed at 0.50m, with a polar angle $\theta_{2}=90^{\circ }$. The convergence time is taken as t=1s.

**Figure 15:**
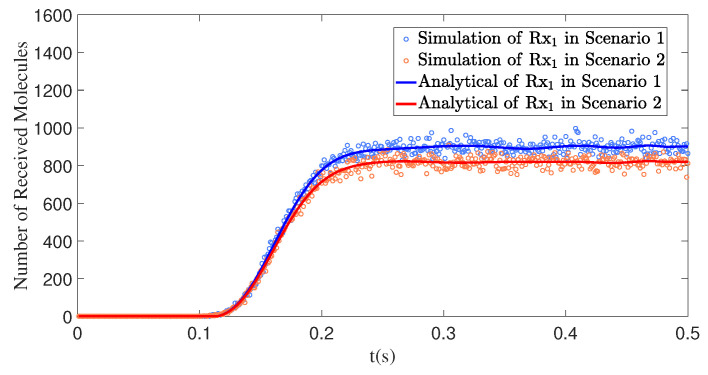
Through model comparison, the number of molecules received by receiver $Rx_{1}$ over time *t* is obtained under different $\phi_{2}$ and $\theta_{2}$. Scenario 1: $d_{1}=1.00\;m,\;\theta_{1}=90^{\circ },\;\phi_{1}=0^{\circ };\;d_{2}=0.50\;m,$ $\theta_{2}=70^{\circ },\;\phi_{2}=20^{\circ }$. Scenario 2: $d_{1}=1.00\;m,\;\theta_{1}=90^{\circ },\;\phi_{1}=0^{\circ };\;d_{2}=0.50\;m,\;\theta_{2}=80^{\circ },\;\phi_{2}=10^{\circ }$.

**Figure 16:**
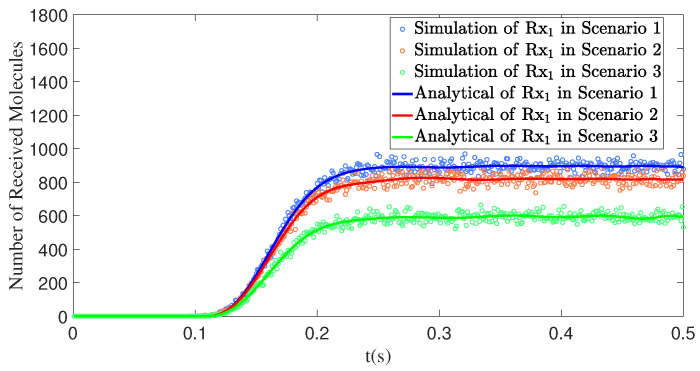
Through model comparison, the number of molecules received by receiver $Rx_{1}$ over time *t* is obtained under different numbers of interfering receivers. Scenario 1: $d_{1}=1.00\;m,\;\theta_{1}=90^{\circ },$ $\phi_{1}=0^{\circ };\;d_{2}=0.65\;m,\;\theta_{2}=80^{\circ },\;\phi_{2}=10^{\circ }$. Scenario 2: $d_{1}=1.00\;m,\;\theta_{1}=0^{\circ },\;\phi_{1}=0^{\circ };$ $d_{2}=0.65\;m,\;\theta_{2}=80^{\circ },\;\phi_{2}=10^{\circ };\;d_{3}=0.50\;m,\;\theta_{3}=70^{\circ },\;\phi_{3}=20^{\circ }$. Scenario 3: $d_{1}=1.00\;m,$ $\theta_{1}=0^{\circ },\;\phi_{1}=0^{\circ };\;d_{2}=0.65\;m,\;\theta_{2}=80^{\circ },\;\phi_{2}=10^{\circ };\;d_{3}=0.50\;m,\;\theta_{3}=70^{\circ },\;\phi_{3}=20^{\circ };$ $d_{4}=0.35\;m,\;\theta_{4}=60^{\circ },\;\phi_{4}=30^{\circ }$.

Figure 13, Figure 14, Figure 15 and Figure 16 illustrate the impact of variations in the polar angle $\theta_{2}$ and azimuthal angle $\varphi_{2}$ of the interfering receiver $Rx_{2}$ on the number of molecules received by the primary receiver $Rx_{1}$. The changes in both the polar and azimuthal angles reflect the occlusion effect of the interfering receiver on the primary receiver as well as its directional characteristics.

In Figure 13a, both scenarios include a primary receiver, $Rx_{1}$, and an interfering receiver, $Rx_{2}$. $Rx_{2}$ influences the number of molecules received by the primary receiver, $Rx_{1}$, by varying the polar angle $\theta_{2}$. When the polar angle $\theta_{2}$ is $80^{\circ }$ (as in Scenario 2), $Rx_{2}$ is positioned directly in front of the transmitter, Tx, receiving a larger number of molecules. This results in a stronger blocking effect on $Rx_{1}$, significantly reducing the number of molecules received by $Rx_{1}$. Conversely, when $\theta_{2}$ is $70^{\circ }$ (as in Scenario 1), $Rx_{2}$ receives fewer molecules, weakening its blocking effect on $Rx_{1}$ and thereby reducing the level of interference. Figure 13b further illustrates the relationship between $Rx_{1}$ and $Rx_{2}$. As $\theta_{2}$ increases, the curve for $Rx_{1}$ exhibits a trend of first decreasing and then increasing within the range of $60^{\circ }<\theta_{2}<120^{\circ }$. This indicates that when $\theta_{2}$ approaches $90^{\circ }$, the blocking effect of $Rx_{2}$ on $Rx_{1}$ reaches its maximum. Outside this angular range, the blocking effect is weaker, and the curve shows smaller fluctuations.

In both scenarios of Figure 14a, a primary receiver $Rx_{1}$ and an interfering receiver $Rx_{2}$ are set up. $Rx_{2}$ affects the number of molecules received by the primary receiver $Rx_{1}$ by altering the azimuth angle $\varphi_{2}$. Similarly, changes in the azimuth angle $\varphi_{2}$ exhibit the same trend. When $\varphi_{2}$ approaches $0^{\circ }$ (e.g., Scenario 2, $\varphi_{2}$ = $0^{\circ }$), $Rx_{2}$ is positioned directly in front of $Rx_{1}$, resulting in the strongest blocking effect on $Rx_{1}$ and the lowest number of molecules received by $Rx_{1}$. Conversely, when $\varphi_{2}$ is larger (e.g., Scenario 1, $\varphi_{2}$ = $70^{\circ }$), the blocking effect of $Rx_{2}$ weakens, and the reception efficiency of $Rx_{1}$ improves [24]. Figure 14b shows the curve relationship between $Rx_{1}$ and $Rx_{2}$ with respect to $\varphi_{2}$. Within the range of ${-30}^{\circ }<\varphi_{2}<30^{\circ }$, the $Rx_{1}$ curve first decreases and then rises, indicating that when $\varphi_{2}$ is close to $0^{\circ }$, $Rx_{2}$ exerts the strongest blocking effect on $Rx_{1}$. In other azimuth angle ranges, the blocking effect is weaker, and the curve fluctuates less.

Overall, the variations in polar angle and azimuth angle both demonstrate the significant directionality of the shielding effect that the interfering receiver exerts on the primary receiver. When the interfering receiver is located directly in front of the main receiver (e.g., $\theta =90^{\circ }$, $\varphi =0^{\circ }$), its shielding effect on the main receiver is the most significant. However, as the interfering receiver deviates from this direction, the reception performance of the main receiver improves. Beyond a certain angular range (e.g., $0^{\circ }<\theta <70^{\circ }$, $110^{\circ }<\theta <180^{\circ }$, ${-90}^{\circ }<\varphi <{-30}^{\circ }$, $30^{\circ }<\varphi <90^{\circ }$), the reception performance of the main receiver stabilizes within a certain range.

Figure 15 illustrates the impact of simultaneously varying the polar angle $\theta_{2}$ and the azimuthal angle $\varphi_{2}$ of the interfering receiver $Rx_{2}$ on the number of molecules received by the primary receiver $Rx_{1}$. When both the polar and azimuthal angles of $Rx_{2}$ increase (Scenario 1), $Rx_{2}$ moves further away from the direct front of the transmitter Tx. Compared to the scenarios in Figure 13 and Figure 14, the distance of $Rx_{2}$ from the x-axis becomes greater, further reducing its blocking effect on $Rx_{1}$ and significantly lowering the level of interference. This further confirms that the more the position of the interfering receiver deviates from the direction of the primary receiver, the weaker its impact on the primary receiver, which helps improve the reception efficiency of the primary receiver.

#### 4.4.3. The Number of Interfering Receivers Influences the Number of Molecules Received by the Primary Receiver

Figure 16 illustrates the impact of the number of interfering receivers on the number of molecules received by the primary receiver, $Rx_{1}$. In Scenarios 1 through 3, apart from the primary receiver $Rx_{1}$, one, two, and three interfering receivers are introduced, respectively. Specifically, Scenario 1 includes only one interfering receiver, $Rx_{2}$, while Scenario 2 adds an additional interfering receiver, $Rx_{3}$, and Scenario 3 further introduces $Rx_{4}$. As the number of interfering receivers increases, the obstructed area between $Rx_{1}$ and the transmitter $Tx_{1}$ expands, reducing the propagation paths available for molecules to reach $Rx_{1}$. Consequently, the number of molecules received by $Rx_{1}$ in Scenario 3 is lower compared to Scenarios 1 and 2. This indicates that the number of interfering receivers has a direct impact on the reception efficiency of the primary receiver; the more interfering receivers there are, the poorer the reception performance of the primary receiver becomes.

In summary, the interplay of angle, distance, and interfering receivers significantly impacts the performance of MC systems. The reception efficiency is highest when the receiver is positioned directly in front of the transmitter and at a close distance, while deviations in angle or increases in distance lead to reduced efficiency. The presence of interfering receivers notably affects the reception efficiency of the primary receiver, with the degree of impact depending on the position and number of interfering receivers. Particularly in multi-receiver scenarios, the occlusion effect caused by interfering receivers can substantially lower the reception efficiency of the primary receiver. In the design of pulse-assisted SIMO MC systems, the relative positions among receivers, the spatial relationship between each receiver and the transmitter, and the number of receivers within the system should be carefully coordinated to avoid excessive interference with the primary receiver. Additionally, the initial velocity of molecules influences their propagation distance and the number of molecules received. System design should also account for the effects of environmental flow velocity and the initial velocity of molecules on system performance, enabling the appropriate control of flow velocity magnitude and direction to improve overall system performance.

## 5. Discussion

Research findings demonstrate that the spatial position of receivers is critical to system performance. When a receiver is situated at a polar angle of 90° and an azimuthal angle of 0°, its reception efficiency is maximized. Any deviation from this optimal angle significantly diminishes reception performance. Moreover, the number of molecules received decreases monotonically with increasing distance between the transmitter and the receiver.

Through in-depth analysis, this study further quantifies the interference effects among multiple receivers. The results indicate that, as the distance between an interfering receiver and the transmitter increases from 0.10 m to 0.95 m, the number of molecules received by the primary receiver first rises and then falls, exhibiting a nonlinear interference pattern. In addition, the study confirms that reception efficiency of the primary receiver declines with an increasing number of interfering receivers. While increasing the initial velocity of the molecules can expand the overall propagation range, it may also lead to reductions in reception rate within specific regions—a trade-off requiring careful consideration in system design.

In practical scenarios (such as within a patient’s body), integrating this model with nanomachines (such as engineered cells) can effectively enable precise detection of human health information. For instance, when a nanomachine identifies an abnormal condition (such as elevated insulin levels) within the body, it rapidly transmits this information to other nanomachines using the communication mechanism of information molecules. The other nanomachines then decode the received information molecules according to the model algorithms and continue relaying the information. The ultimate nanorouter acts as a center for information integration and forwarding; upon receiving information from various nanomachines, it transmits the health data to medical devices via wireless signals. This process not only facilitates efficient real-time monitoring but also provides timely feedback to healthcare professionals, aiding in rapid decision-making and intervention.

However, there are still some limitations in our study that need to be clearly addressed. First, our model assumes an ideal spherical receiver for absorption; however, in actual nanoparticle communication, the absorption efficiency of the receiver and saturation effects cannot be ignored. Incomplete absorption may lead to some signals not being effectively captured by the receiver, thereby reducing signal strength and affecting decoding capability. Additionally, unabsorbed signals increase the noise-to-signal ratio, which may raise the bit error rate and diminish the overall reliability of the system. When the receiver reaches a saturation state, its response to the signal will no longer increase, leading to signal distortion and limiting the dynamic range. Secondly, in actual biological environments, molecules are not permanently stable and can gradually degrade or lose activity due to various enzymes and chemical reactions. If this degradation process is not incorporated into the model, the calculated molecular concentration may be overestimated. Finally, under controlled laboratory conditions, the release rate and duration are typically set to ideal states; however, in real biological systems, these conditions may be influenced by fluctuating environmental factors. For instance, factors such as pH, temperature, and ionic strength can significantly affect the release rate and effectiveness of the molecules. We will delve deeper into the quantitative effects of these factors in future research.

## 6. Conclusions

In this study, a pulse-assisted SIMO MC system model is established in three-dimensional space. By comprehensively considering fluid resistance and convection velocity, and introducing a fixed-direction pulsed airflow, the model more accurately captures molecular propagation characteristics in biological settings. Drawing on the convection–diffusion equation and Stokes’ law, the model systematically integrates factors such as fluid resistance and convection velocity.

## Figures and Tables

**Table 1:** Comparison of different model characteristics.

Model	Attribute
*SISO Model*	**Fluid Velocity Effects:** Partially considered in some studies. Velocity directed toward the receiver. **Interference Among Receivers:** Not applicable. **Application Scenarios:** Free-diffusion and fluid-assisted scenarios. **Maximum Model Dimension:** Three-dimensional. **Factors Influencing System Performance:** Distance.
*SIMO Model*	**Fluid Velocity Effects:** Not addressed. **Interference Among Receivers:** Addressed in limited studies **Application Scenarios:** Free-diffusion scenario. **Maximum Model Dimension:** Mostly two-dimensional. Some three-dimensional studies simplified to two-dimensional. **Factors Influencing System Performance:** Distance. If receiver interference is studied, also consider receiver–receiver and receiver–transmitter angles.
*Our Fluid-Assisted* *SIMO Model*	**Fluid Velocity Effects:** Considers fluid resistance and convection velocity. **Interference Among Receivers:** Quantifies the interference effects among multiple receivers. **Application Scenarios:** Free-diffusion and fluid-assisted scenarios. **Maximum Model Dimension:** Three-dimensional. **Factors Influencing System Performance:** Distance, initial fluid velocity. If receiver interference is studied, also consider the polar and azimuthal angles of receiver–receiver and receiver–transmitter.

**Table 2:** Simulation parameters.

Parameter	Value
Number of transmitting molecules per step (*N*)	3000
Dynamic viscosity of fluid (η)	$8.9\times 10^{-4}\;\mathrm{kg}{\left(\mathrm{ms}\right)}^{-1}$
Radius of molecules (*r*)	$50\times 10^{-6}\;m$
Initial velocity ($u_{0}$)	$1.50\;ms^{-1}$
Mass of molecules (*m*)	$6\times 10^{-10}\;\mathrm{kg}$
Diffusion coefficient (*D*)	$10^{-5}\;m^{2}s^{-1}$
Radius of receiver (*R*)	$2\times 10^{-2}\;m$
Simulation step (Δt)	$10^{-3}\;s$
Simulation duration	2s
Simulation repetitions	100

## Data Availability

Not applicable.

## Appendix A

### Appendix A.1

In this section, we will present a detailed derivation from Equations (8)–(10). For the flow term in Equation (8), we introduce a special linearization to eliminate the flow term. Defining the new variable $\xi =x-s\left(t\right),\;\mathrm{where}\;s\left(t\right)=\int_{0}^{t}u\left(\tau \right)d\tau ,$ we can thus apply the chain rule:

(A1) $$\frac{\partial C}{\partial t}=\frac{\partial \hat{C}}{\partial t}-u\left(t\right)\frac{\partial \hat{C}}{\partial \xi },\;\frac{\partial C}{\partial x}=\frac{\partial \hat{C}}{\partial \xi }.$$

Substituting the replaced expression into the original equation, we obtain

(A2) $$\frac{\partial \hat{C}}{\partial t}-D\nabla^{2}\hat{C}=N\delta \left(\xi +s\left(t\right)\right)\delta \left(y\right)\delta \left(z\right)\delta \left(t\right),$$

where $\nabla^{2}=\frac{\partial^{2}}{\partial x^{2}}+\frac{\partial^{2}}{\partial y^{2}}+\frac{\partial^{2}}{\partial z^{2}}.$ The source term δ(ξ+s(t)) indicates that the location of the source varies over time, requiring further processing. For t>0, s(t) is a monotone increasing function, allowing us to perform the variable substitution τ=s(t). To solve the nonlinear expansion procedure, we employ a Galerkin method, which requires

(A3) $$\frac{\partial G}{\partial t}-D\nabla^{2}G=\delta \left(\xi \right)\delta \left(y\right)\delta \left(z\right)\delta \left(t\right).$$

Applying the Laplace transform with respect to time *t*,

(A4) $$L\left\{G\left(\xi ,y,z,t\right)\right\}=\tilde{G}\left(\xi ,y,z,s\right),\;L\left\{\frac{\partial G}{\partial t}\right\}=s\tilde{G}\left(\xi ,y,z,s\right)-G\left(\xi ,y,z,0\right).$$

At t=0, since s(0)=0, it follows that G(ξ,y,z,0)=0. Therefore, the boundary condition is

(A5) $$s\tilde{G}-D\nabla^{2}\tilde{G}=\delta \left(\xi \right)\delta \left(y\right)\delta \left(z\right).$$

For the spatial variables ξ,y,z, a three-dimensional Laplace transform is applied:

(A6) $$F\left\{\tilde{G}\left(\xi ,y,z,s\right)\right\}=\hat{G}\left(k_{\xi },k_{y},k_{z},s\right),\;F\left\{\delta \left(\xi \right)\right\}=1.$$

Using the properties of the Fourier transform,

(A7) $$F\left\{\nabla^{2}\tilde{G}\right\}=-\left(k_{\xi }^{2}+k_{y}^{2}+k_{z}^{2}\right)\hat{G}=-k^{2}\hat{G},\;k^{2}=k_{\xi }^{2}+k_{y}^{2}+k_{z}^{2}.$$

The equation transforms to

(A8) $$s\hat{G}+Dk^{2}\hat{G}=1⟹\hat{G}\left(k_{\xi },k_{y},k_{z},s\right)=\frac{1}{s+Dk^{2}}.$$

The three-dimensional Fourier inverse transform yields

(A9) $$\tilde{G}\left(\xi ,y,z,s\right)=\frac{1}{{\left(2\pi \right)}^{3}}\;\int \int \int \;\frac{\exp\left(i(k_{\xi }\xi +k_{y}y+k_{z}z)\right)}{s+Dk^{2}}\;dk_{\xi }\;dk_{y}\;dk_{z}.$$

Convert the following to a spherical coordinate system $\left(k_{\xi }=k\sin\theta \cos\varphi ,k_{y}=k\sin\theta \sin\varphi ,k_{z}=k\cos\theta \right)$

(A10) $$\tilde{G}\left(\xi ,y,z,s\right)=\frac{1}{{\left(2\pi \right)}^{3}}\int_{0}^{2\pi }d\varphi \int_{0}^{\pi }d\theta \int_{0}^{\infty }\frac{\exp(ikr\cos\theta )}{s+Dk^{2}}k^{2}\sin\theta \;dk,$$

where $r=\sqrt{\xi^{2}+y^{2}+z^{2}}$. After integration, the following can be obtained:

(A11) $$\tilde{G}\left(\xi ,y,z,s\right)=\frac{1}{4\pi Dr}\exp\left(-\sqrt{\frac{s}{D}}r\right).$$

From the inverse Laplace transform table,

(A12) $$L^{-1}\left\{\frac{\exp\left(-\sqrt{\frac{s}{D}}r\right)}{r}\right\}=\frac{1}{\sqrt{4\pi Dt^{3}}}\exp\left(-\frac{r^{2}}{4Dt}\right).$$

Thus,

(A13) $$G\left(\xi ,y,z,t\right)=\frac{1}{4\pi Dr}\cdot \frac{r}{\sqrt{4\pi Dt^{3}}}\exp\left(-\frac{r^{2}}{4Dt}\right)=\frac{1}{{\left(4\pi Dt\right)}^{3/2}}\exp\left(-\frac{\xi^{2}+y^{2}+z^{2}}{4Dt}\right).$$

This indicates how the concentration diverging from the source point changes over time. By applying the superposition integral method, we can obtain the desired concentration:

(A14) $$\hat{C}\left(\xi ,y,z,t\right)=N\int_{0}^{t}G\left(\xi +s\left(\tau \right),y,z,t-\tau \right)\delta \left(\tau \right)d\tau =NG\left(\xi +s\left(0\right),y,z,t\right).$$

Since at t=0, s(0)=0, we can simplify

(A15) $$\hat{C}\left(\xi ,y,z,t\right)=\frac{N}{{\left(4\pi Dt\right)}^{3/2}}\exp\left(-\frac{\xi^{2}+y^{2}+z^{2}}{4Dt}\right).$$

Finally, let ξ=x−s(t) be a substitution variable; we then obtain Equation (10).

### Appendix A.2

In the current model, the flow direction is set along the x-axis primarily because, in real scenarios, most flows occur in the x-direction, such as blood flow in human vessels, the transmission of plant signaling substances, and the diffusion of liquids. However, in practical applications, the model possesses significant flexibility to accommodate arbitrary flow directions. Mathematically, the treatment for the y- and z-axes can be made analogous to that for the x-axis. Assuming the flow velocity is $\vec{v}=\left[v_{x},v_{y},v_{z}\right]$, the corresponding position evolution is given by $\vec{s}\left(t\right)=\left[s_{x}\left(t\right),s_{y}\left(t\right),s_{z}\left(t\right)\right]$, where $\vec{s}\left(t\right)=\left[s_{x}\left(t\right),s_{y}\left(t\right),s_{z}\left(t\right)\right]$ represents the position of the fluid in the x-, y-, and z-directions over time, respectively. The resulting concentration distribution is given by

(A16) $$C\left(x,y,z,t\right)=\frac{N}{{\left(4\pi Dt\right)}^{3/2}}\exp\left(-\frac{{\left(x-s_{x}\left(t\right)\right)}^{2}+{\left(y-s_{y}\left(t\right)\right)}^{2}+{\left(z-s_{z}\left(t\right)\right)}^{2}}{4Dt}\right).$$

However, the actual displacement equations need to be derived based on the force analysis corresponding to the relevant directions.
